# Adapting a Pain Coping Skills Training Intervention for Patients with Dementia and their Caregivers

**DOI:** 10.1093/geroni/igaf122.2571

**Published:** 2025-12-31

**Authors:** Laura Porter, Katherine Ramos, Francis Keefe, Deborah Weiner, Heather Whitson, Courtney Van Houtven, Natalie Leary

**Affiliations:** Duke University Medical Center, Durham, North Carolina, United States; Duke University School of Medicine, Durham, North Carolina, United States; Duke University School of Medicine, Durham, North Carolina, United States; University of Pittsburgh, Pittsburgh, Pennsylvania, United States; Duke University, Durham, North Carolina, United States; Duke University, Durham, North Carolina, United States; Duke University School of Medicine, Durham, North Carolina, United States

## Abstract

Community dwelling persons with dementia (PWD) and their family caregivers represent a burgeoning and vulnerable population with significant unmet needs, one of which is pain management. Pain in PWD is common, causes increased distress and disability for patients, and increased stress for caregivers. Pain coping skills training (PCST) is a non-pharmacological pain management approach based on cognitive-behavioral principles that is efficacious among older adults without dementia. However, little is known about how to adapt PCST to meet the unique needs of PWD and their caregivers. In this study, we recruited six PWD (50% female, 33% Black, 77% White) and their caregivers (83% female, 67% spouses, 33% Black, 77% White) to form a Community Advisory Board (CAB). We met with them three times to obtain their input on intervention delivery and content. CAB members responded favorably to the proposed intervention length (five 45-minute sessions) and delivery to patient-caregiver dyads. They primarily preferred participation via videoconference. We presented four pain coping skills (PAINAD tool for pain assessment, relaxation, coping thoughts, and pleasant activity scheduling). Most CAB members reported perceiving the skills as highly useful, although there was variation based on the PWD’s level of cognitive impairment (mild-moderate). They provided valuable input on (a) ways to tailor content and delivery for varying levels of cognitive impairment, and (b) materials (e.g., audio recordings, handouts) to promote learning and practice of skills. This feedback will be used to refine the intervention before implementing a single-arm pilot trial with 30 dyads.

